# Biological Restorations: An Alternative Esthetic Treatment for Restoration of Severely Mutilated Primary Anterior Teeth

**DOI:** 10.5005/jp-journals-10005-1008

**Published:** 2008-12-26

**Authors:** Grewal N, Reeshu S

**Affiliations:** 1 Professor and Head, Department of Pedodontics and Preventive Dentistry, Government Dental College Amritsar, Punjab, India, e-mail: navgredent@hotmail.com; 2 Postgraduate student, Department of Pedodontics and Preventive Dentistry, Government Dental College Amritsar, Punjab, India

**Keywords:** Early childhood caries, biological restorations.

## Abstract

Early childhood caries (ECC) affects more than one out of seven preschoolers and is more prevalent in lowincome families, who generally have limited access to dental services. The seriousness and societal costs of ECC continue to be a significant health issue for children from racial/ethnic minorities and from developing countries. Hence, a *biological restoration* seems to be a successful cost-effective alternative approach for treating such cases.

## INTRODUCTION

Jonathan Kozol^1^ , said that ECC (Early Childhood Caries) is a grave public health problem which exists in our most vulnerable citizens –‘Our Children.’ Although dental caries has been declining globally in general population ,its prevalence in young children has not shown a significant decline.

Mahejabeen R, et al (2006)^2^ studied the prevalence of caries in 1500 children aged 3-5 years in the Dharwad district of Karnataka, India. The overall prevalence was found to be 54.1%. The caries pattern revealed that 23% children had caries in anterior teeth only.

Most frequent presentation of ECC is severely mutilated primary anterior teeth. Restoring these is a challenging job for the pedodontist and over the years many clinicians have tried various procedures to restore them. In cases of severe loss of tooth structure intra-canal posts become mandatory. Recent developments in restorative materials, placement techniques and adhesive protocols facilitate these restorations.^3^-^5^ However ,these procedures turn out to be expensive and technique sensitive and also require expertise of operator.

A biological restoration^6^ meets up to the esthetic and structural standards of natural teeth. They provide natural posts and crowns which can fit into the treated root stumps of the individual and replace the coronal portion esthetically. This case report shows the success of placing biological restorations in children with severely mutilated primary anterior teeth.

## CASE 1

**Fig. 1:**
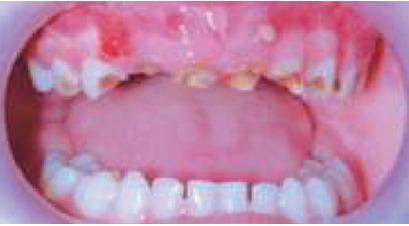
Abscess formation in relation to 51,61

A 4-year-old girl child reported to the Department of Pedodontia and Preventive Dentistry, Government Dental College Amritsar, with chief complaint of decayed anterior teeth. On examination it was found that all the primary anterior teeth were carious and only root stumps were seen. There was associated pain and infection showing abscess formation in relation to 51, 52 and 61 (Fig. 1). Besides carious involvement in the anterior region there were proximal and buccal lesions in the maxillary canines and first molars. A detailed history of feeding habits revealed history of bottle feeding till the age of 3 years. Oral hygiene practices involved brushing once daily sometimes without toothpaste.

The loss of coronal portion had affected the speech articulation of the child. She was shy and the parents reported an introvert social behavior. After complete discussion of treatment options with the parents the parents opted for biological restoration procedure because of cost effectiveness since four anteriors required to be restored.

**Fig. 2:**
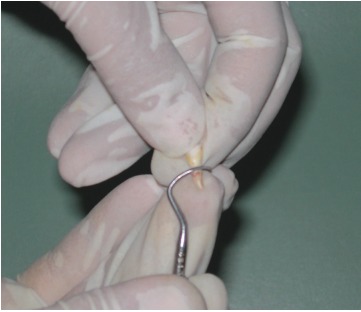
Removal of soft tissue and PDL remnants from the root surface

**Fig. 3:**
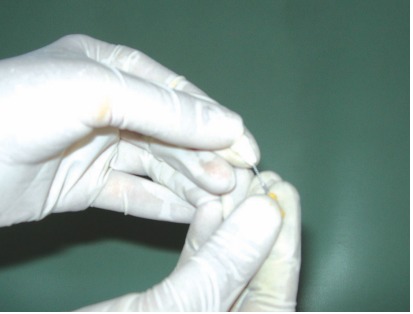
Extirpation of pulpal tissue and remnants from root canal

**Fig. 4:**
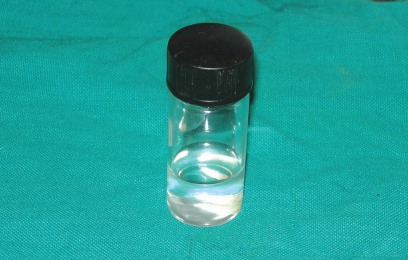
Prepared and sterilized tooth stored in Hank’s balanced salt solution

Complete consent form records of the donor and recipient were maintained in the tooth bank records of the Department. The selected samples were procured from the Tooth Tissue Bank maintained in the Department of Pedodontics, GDC. Amritsar, where they are stored and sterilized after thorough scaling and removal of soft tissue, periodontal remnants and pulpal tissue from the root-canals and kept at 4 degree centigrade in Hank’s balanced salt solution with donor identification^7^-^9^ (Figs 2 to 4).

### Endodontic Preparation

**Fig. 5:**
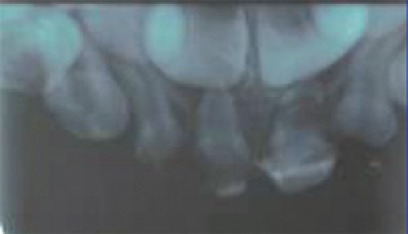
Preoperative radiograph

**Fig. 6:**
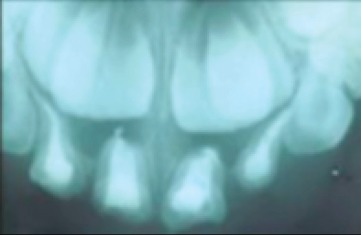
Postoperative radiograph

**Fig. 7:**
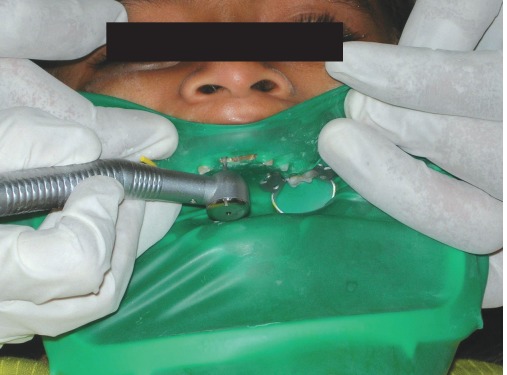
Removal of endodontic filling material for preparation of core to take up the biological-post

The endodontic treatment of the carious teeth was carried out using NiTi files and reamers and after complete sterilization of canals obturation with Vitapex was done. The obturating material in the coronal one third of the root was removed and the root prepared to receive intracanal portion of the biological post (Figs 5 to 7).

### Preparation of the Biological Restoration (Figs 8 and 9)

Teeth selected from the tooth bank were reshaped with a crown preparation kit and the roots shaped to function as posts. The apical third was removed and the remaining root tump filled retrograde with flowable composite material. The shaped tooth was then tried for fit in the prepared root canal and readjusted for a snug fit. The finally prepared crown and root were cemented with dual cure resin modified GIC. Excess extruded material was finished and polished to give a final esthetic result as shown in Figures 10A and B.

**Fig. 8:**
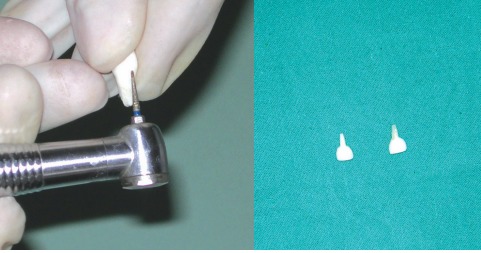

**Fig. 9:**
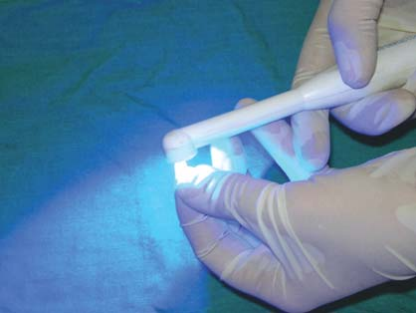


Figs 8 and 9: Preparation and retrograde filling of root-stump with flowable composite

**Figs 10A and B:**
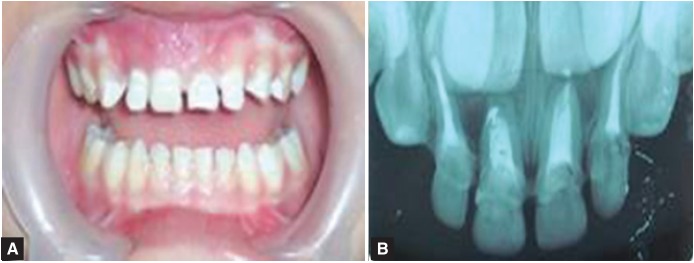
Final esthetic result

### Post-treatment Follow-up

**Figs 11:**
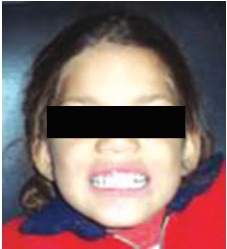
Showing retentivity of biological post after 12 months

The restorations were evaluated for any marginal discoloration, loss of restoration, color change, recurrent caries and retention after 3 months, 6 months, 9 months and 12 months respectively (Fig. 11). The gingival health was visually and clinically inspected with explorer and rated. The assessment of patients response to treatment indicated positive change in behavior, speech and self esteem.

## CASE 2

A Three-year-old male child reported with ECC and some previously done restorations in 51, 52, 61 and 62. The composite restoration in 62 was lost and the parents opted for a biological restoration. The procedure was followed as described earlier and the completed restoration is shown in Figures 12A to E.

## CASE 3

A four-year-old female child reported with pain on biting in upper anterior region and was repeatedly put on antiinflammatory and analgesic medication since last 6 to 7 months. After completing endodontic procedures in 51, 61.

The patient was given the option of biological restorations as shown in Figures 13A to F respectively.

## DISCUSSION

This method of using biological crown and post restoration for primary teeth affected by ECC has shown promising results. The cost of these restorations when compared with conventional methods of using intracanal reinforced composite resin restorations was six to seven times lesser. Hence it proved to be a cost effective alternative making it possible to recycle precious biological tissue which has been discarded as bio-waste.

However, patient acceptance of a biological restoration is an important issue and donor selection from siblings could be a more acceptable alternative. The Human tooth bank is a nonprofit institution associated with the department of Pedodontics and preventive Dentistry in the postgraduate research section which fulfils academic needs of research scholars. In order to work ethically a universal protocol as established in Univ. of Sao Paulo Brazil has been followed in structuralizing the tooth-bank.^8^

**12A to E:**
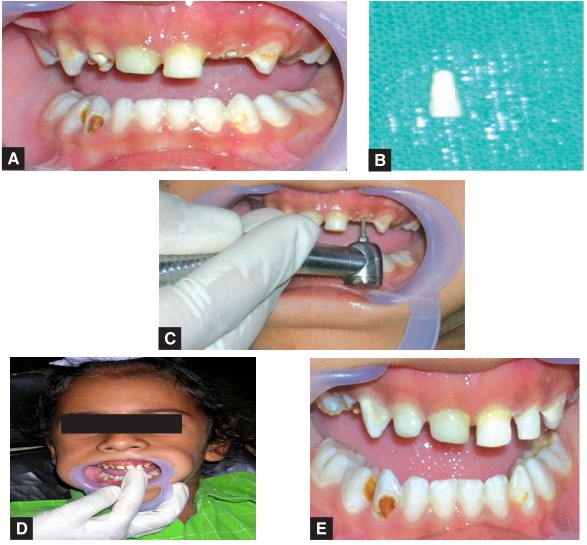
Biological restoration given in previously lost composite buildup in 62

**13A to F:**
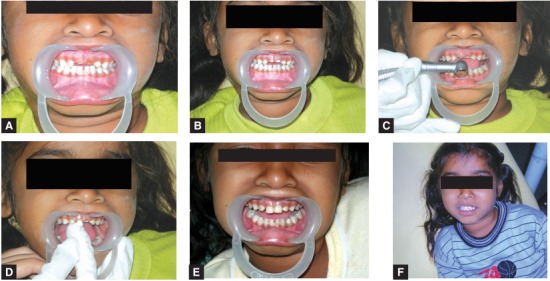
The same patient after 15 months
